# A Lab-Made E-Nose-MOS Device for Assessing the Bacterial Growth in a Solid Culture Medium

**DOI:** 10.3390/bios13010019

**Published:** 2022-12-24

**Authors:** Teresa Dias, Vítor S. Santos, Tarek Zorgani, Nuno Ferreiro, Ana I. Rodrigues, Khalil Zaghdoudi, Ana C. A. Veloso, António M. Peres

**Affiliations:** 1 Centro de Investigação de Montanha (CIMO), Instituto Politécnico de Bragança, Campus de Santa Apolónia, 5300-253 Bragança, Portugal; 2 Laboratório Associado para a Sustentabilidade e Tecnologia em Região de Montanha (SusTEC), Instituto Politécnico de Bragança, Campus de Santa Apolónia, 5300-253 Bragança, Portugal; 3 Departamento de Medicina Veterinária, Universidade Federal de Mato Grosso, Campus Sinop, Avenida Alexandre Ferronato, nº 1200, Bairro Residencial Cidade Jardim, Sinop 78550-728, MT, Brazil; 4 Département Génie Chimique, Université Libre de Tunis, Avenue Khéreddine—Pacha Tunis, 30, Tunis 1002, Tunisia; 5 Instituto Politécnico de Coimbra, ISEC, DEQB, Rua Pedro Nunes, Quinta da Nora, 3030-199 Coimbra, Portugal; 6 CEB—Centre of Biological Engineering, University of Minho, Campus de Gualtar, 4710-057 Braga, Portugal; 7 LABBELS–Associate Laboratory, 4800-058 Braga/Guimarães, Portugal

**Keywords:** electronic nose, metal oxide semiconductor sensors, bacteria identification, Gram-positive, Gram-negative, colony-forming units, linear discriminant analysis, multiple linear regression models, simulated annealing algorithm

## Abstract

The detection and level assessment of microorganisms is a practical quality/contamination indicator of food and water samples. Conventional analytical procedures (e.g., culture methods, immunological techniques, and polymerase chain reactions), while accurate and widely used, are time-consuming, costly, and generate a large amount of waste. Electronic noses (E-noses), combined with chemometrics, provide a direct, green, and non-invasive assessment of the volatile fraction without the need for sample pre-treatments. The unique olfactory fingerprint generated during each microorganism’s growth can be a vehicle for its detection using gas sensors. A lab-made E-nose, comprising metal oxide semiconductor sensors was applied, to analyze solid medium containing Gram-positive (*Enterococcus faecalis* and *Staphylococcus aureus*) or Gram-negative (*Escherichia coli* and *Pseudomonas aeruginosa*) bacteria. The electrical-resistance signals generated by the E-nose coupled with linear discriminant analysis allowed the discrimination of the four bacteria (90% of correct classifications for leave-one-out cross-validation). Furthermore, multiple linear regression models were also established allowing quantifying the number of colony-forming units (CFU) (0.9428 ≤ *R*^2^ ≤ 0.9946), with maximum root mean square errors lower than 4 CFU. Overall, the E-nose showed to be a powerful qualitative–quantitative device for bacteria preliminary analysis, being envisaged its possible application in solid food matrices.

## 1. Introduction

The early and fast detection of foodstuffs’ contamination with pathogenic bacteria is of major relevance and can be used as a quality and/or safety diagnosis tool throughout the entire food chain. Conventional culture-based methods, immunological assays and polymerase chain reaction methods are widely used as reliable, specific, and sensitive standard techniques despite being time-consuming, expensive, labor-intensive technicians, and being not suitable as point-of-use sensing devices [1,2,3].

Alternative strategies for microorganisms’ screening (e.g., fungi, bacteria, etc.) consider the detection of specific volatile organic compounds (VOCs) generated during the microorganisms’ primary and secondary metabolisms, like alcohols, aldehydes, hydrocarbons, acids, ethers, esters, ketones, and terpenes [3]. Thus, gas chromatography coupled with mass spectrometry (GC-MS) is the standard analytical technique, allowing identifying and quantifying the volatiles related to each microorganism [4,5,6,7,8,9]. During their growth, each microorganism emits characteristic volatile compounds, generating distinctive odor fingerprints, which can be used for discrimination purposes [10]. The volatile pattern and amounts released by each microorganism may differ with the strain, incubation time, and growth conditions (i.e., substrate, nutrients, pH, humidity, and temperature) [3,11]. However, different bacteria can produce the same VOCs during their growth (ammonia is emitted by *Pseudomonas aeruginosa* or *Staphylococcus aureus*; methanol, 1-propanol, 1-butanol, and indole are associated with *Escherichia coli*; and acetoin and diacetyl with *Enterococcus faecalis*) [4,5,6,7,8,9,10,11].

In this sense, the use of electronic noses (E-noses), capable of encoding high-dimension VOCs’ patterns into a smaller-dimension pattern of sensors signals, has emerged as a practical and feasible non-invasive alternative for detecting bacteria, as recently reviewed by Bonah et al. [10]. Different gas sensors have been used namely, metal oxide (MOX) or metal oxide semiconductors (MOS), and polymer sensors. To extract the information from the signals profiles, different chemometric tools have been applied, like principal component analysis (PCA), hierarchical cluster analysis (HCA), linear discriminant analysis (LDA), uncorrelated LDA (ULDA), and/or support vector machines (SVM). E-noses coupled with multivariate statistical techniques, have been successfully used for bacteria detection (e.g., *Bacillus cereus*, *E. coli*, *E. faecalis*, *Listeria innocua*, *P. aeruginosa*, *S. aureus*, or *Salmonella* spp.) in different culture media or foods [10]. It should be remarked that, besides this particular type of application, the use of E-noses in food analysis has been widely exploited for different matrices [12,13,14].

Regarding microorganisms’ evaluation, it was possible to discriminate (predictive accuracies from 84 to 97.5%) different bacteria (*B. cereus*, *E. coli*, *E. faecalis*, *Listeria* spp., and/or *Salmonella* spp.) grown in solid or liquid synthetic media using E-nose-MOS devices coupled with ULDA or SVM models [1,2,15,16]. It was possible to discriminate viable from non-viable bacteria (*E. coli* and *Yersinia enterocolitica*) using an E-nose-MOS-LDA model (predictive accuracies of 100%) grown in a liquid synthetic medium or in skimmed milk samples [17]. Other researchers also showed that an E-nose-MOS coupled with discriminant function analysis or probabilistic neural network could discriminate among *E. coli*, *P. aeruginosa*, and *Klebsiella oxytoca* in water samples (predictive accuracy of 87% [18]. Commercial E-noses (Cyranse 320 from Cyrano Sciences; BH-114, from Bloodhound; or eNOSE 4000, from Marconi Applied Technology), comprising arrays of polymer sensors, allowed the correct classification (98%) of different bacteria (*S. aureus*, *Haemophilus influenza*, *Streptococcus pneumonia*, *E. coli*, *P. aeruginosa*, and *Moraxella catarrhalis*) [19] or to differentiate control samples from those containing *E. coli* or *P. aeruginosa* [20]. Similarly, a hybrid E-nose with organic–inorganic nanocomposite sensors plus MOS sensors was proposed to differentiate *Enterobacter cloacae*, *S. aureus*, *E. coli*, *P. aeruginosa*, and *S. enterica*, grown in a synthetic medium [21]. Recently, an E-nose with carbon-dots was developed allowing, in combination with PCA, to differentiate *E. coli*, *P. aeruginosa*, *S. aureus*, and *B. subtilis*, also grown in synthetic medium [22]. An E-nose-MOX allowed differentiating *E. coli*, *Klebsiella pneumonia*, and *Salmonella enterica* in pasteurized milk samples, with a predictive classification rate of 95% [23]. Optical E-noses were also applied to detect and differentiate (using PCA and/or HCA) different bacteria grown in synthetic media or in drinking water samples (*E. aerogenes*, *E. coli*, *E. faecalis*, *Klebsiella pneumonia*, *Listeria monocytogenes*, *Proteus mirabilis*, *P. aeruginosa*, *Streptococcus agalactiae*, and/or *S. aureus*) [24,25].

However, fewer studies reported the use of E-noses for bacteria quantification. Carrillo-Gómez et al. [23,26] verified the feasibility of using an E-nose-MOX device to semi-quantitatively assess the contamination levels of milk samples [23] or drinking water [26] with *E. coli*. Also, Tonezzer et al. [27] showed that an E-nose, with a single nanowire gas sensor, could accurately assess the total viable count (TVC) during trout fish spoilage, allowing estimating the decimal logarithmic of TVC with an error lower than 5%, using an SVM model. Similarly, an E-nose-MOX device allowed for the predicting of the TVC of aerobic bacteria in sardines, using partial least squares models (correlation coefficient of 0.91), which accuracy was attributed to the capacity of the device to assess the emission of VOCs generated from the sardines’ bacterial degradation [28].

In this sense, the present study aims to evaluate not only the classification, but also the quantification performances of a lab-made E-nose, with commercial MOS gas sensors, for bacteria evaluation, grown in solid media. Four target microorganisms were selected taking into account their use as quality/contamination indicators for food/water samples, namely, two Gram-positive (*E. faecalis* and *S. aureus*) and two Gram-negative (*E. coli* and *P. aeruginosa*) bacteria. Previously, the research team has demonstrated that a potentiometric electronic tongue, comprising lipid polymeric sensor membranes could be used for these purposes, although it required the resuspension of each bacterium dried mass in water, which does not allow a direct analysis [29]. Alternatively, the E-nose-MOS device, coupled with LDA or multiple linear regression models (MLRM), could be used as a green, direct (i.e., not requiring any sample’s pre-treatment) and non-invasive recognition/counting sensing platform for sniffing bacteria growth in solid media.

## 2. Materials and Methods

### 2.1. Bacterial Strains and Inoculum Preparation

Two Gram-positive spherical-shaped (*S. aureus* ATCC653 and *E. faecalis* ATCC29212) and one Gram-negative rod-shaped (*P. aeruginosa* ATCC15442) bacteria were selected as target food-borne pathogens. *E. coli* ATCC29998 was also included in this study since it is a hygiene/fecal indicator. As previously described [29], the *inocula* were prepared by mixing 300 µL of glycerol (Sigma-Aldrich, St. Louis, MO, USA) with 700 µL of an overnight bacterial culture. The growth was promoted in Brain Heart Infusion (BHI) (PanReac AppliChem, ITW Reagents, Barcelona, Spain) at 37 °C, under orbital agitation at 90 rpm (Orbital incubator S1500, Stuart). Each *inoculum* was cryopreserved at −20 °C until being used.

### 2.2. Growth Conditions

Each strain was inoculated aseptically in Erlenmeyer flasks (250 mL) containing 50 mL of BHI medium. Bacterial cultures were grown at 37 °C under orbital agitation (90 rpm) for 24 h until the cells’ stationary phase was reached. After that, the optical density was measured, at 560 nm, in a spectrophotometer (UV-3100 PC Spectrophotometer, VWR). According to preliminary assays (data not shown), sets of pre-established serial dilutions were performed from overnight grown cultures, using NaCl solutions (0.9% *v*/*v*): from 10^−1^ to 10^−8^ for *E. faecalis*; and, from 10^−1^ to 10^−9^ for *P. aeruginosa*, *S. aureus*, and *E. coli*. Colony forming units (CFU) were assessed as follows: 100 µL of each dilution was plated on BHI agar, in duplicate. After overnight incubation at 37 °C, CFUs were counted. Gram staining was carried out to verify the nonexistence of culture contamination, as described by Smith and Hussey [30].

### 2.3. HS-SPME-GC-MS Evaluation of VOCs Emitted by Each Bacteria

The profiles of VOCs emitted by the four bacteria studied, which were separately grown, were established by headspace solid-phase microextraction (HS-SPME) and GC-MS. The volatile fraction was chromatographically evaluated by analyzing the HS of glass vials (50 mL, Duran, Germany) containing 10 mL of BHI agar, previously inoculated with 100 μL of a diluted overnight culture (10^−4^ dilution for *E. faecalis* and 10^−5^ for the others bacterial strains). The vials were sealed with polytetrafluroethylene/silicone screw caps and incubated at 37 °C for 24 h. The HS of the non-inoculated BHI culture medium was also analyzed and used as negative control. A Shimadzu GC-2010 Plus chromatograph with a mass spectrometer Shimadzu GC-MS-QP2010 SE detector was used. Each vial, inoculated with a pre-established amount of one of the four bacteria, was spiked with an accurate amount of 5 μL of the internal standard (IS: α-pinene, 98% from Sigma Aldrich, St. Louis, MO, USA) methanolic solution, with a concentration of 0.50225 mg/mL, being the volatiles adsorbed into an SPME fiber coated with divinylbenzene/carbonex/polydimethylsiloxane (DVB/CAR/PDMS 50/30 μm, from Supelco, Bellefonte, PA, USA). The vials were conditioned for 5 min at 37 °C to allow the release of the VOCs. After this time period, the SPME fiber was exposed for 30 min, at 37 °C, allowing the adsorption of the volatile compounds present in the headspace. The volatile fraction of the solid medium not inoculated with any of the four bacteria was also studied following the same procedure and after being spiked with the same amount of IS. The procedure was repeated twice for each bacterium and not inoculated medium.

As previously described [31], peaks’ separation was accomplished on a TRB-5MS (30 m × 0.25 mm × 0.25 µm) column (Teknokroma, Spain). The injector was set at 220 °C and the manual injections were made in splitless mode. The mobile phase consisted of helium (Praxair, Portugal) at a linear speed of 30 cm/s and a total flow of 24.4 mL/min. The oven gradient temperature was as follows: 40 °C/1 min; 2 °C/min until 220 °C (30 min). The ionization source was maintained at 250 °C with an ionization energy of 70 electronvolts (eV) and an ionization current of 0.1 kilovolts (kV). All mass spectra were acquired by electron ionization, being the spectra fragments identified by comparing with the mass spectra’s databases of the NIST SRD-69 Library (National Institute of Standards and Technology, Gaithersburg, MD, USA) and with the free chemical structure/information databases of PubChem and ChemSpider. The minimum similarity for identification purposes was set equal to 85%. Furthermore, the peaks’ identification was further checked using Kovat’s retention indices. The areas of the chromatographic peaks were determined by integrating the re-constructed chromatogram from the full scan chromatogram using the ion base (m/z intensity 100%) for each compound. For semi-quantification purposes, the amounts of the identified volatiles were calculated by the ratio of each base ion peak area to the area of the internal standard base ion peak area, without considering the response factors, and converted to mass equivalents based on the internal standard concentration used.

### 2.4. E-Nose Setup

#### 2.4.1. Apparatus

The lab-made E-nose used was previously described in detail by the research team [32,33]. Briefly, the device included a sampling heated unit (~30 °C), where up to 4 glass sampling vials (~25 mL) could be placed. Each vial was closed with a screw cap connected to a plastic gas valve allowing, when open, to collect and deliver the gas headspace to the sensing heated unit (~40 °C) by means of a diaphragm vacuum mini pump. The vacuum was monitored using a mini Dial Air Vacuum Pressure Gauge Meter Digital Manometer. The sensing unit comprised 9 commercial MOS Figaro gas sensors (S1: TGS 2600 B00; S2: TGS 2602; S3: TGS 2610 C00; S4: TGS 2611 C00; S5: TGS 2610 D00; S6: TGS 2610 E00; S7: TGS 2612; S8: TGS 826 A00; and S9: TGS 823 C12N; specifications can be found at https://www.figarosensor.com/product/sensor/, accessed on 6 October 2022), which are sensitive towards alcohols, hydrocarbons, hydrogen, carbon monoxide, ammonia, hydrogen sulfide, among other gases.

#### 2.4.2. Sampling and Analysis

At the first use, the sensitive materials of the MOS sensors were activated for 48 h, allowing them to reach the operating sensing temperature (>200 °C). In all subsequent assays, it was only required to heat the sampling and sensing units (~30 min) and then to clean the system by pumping filtered air, before testing each sample. A vacuum environment of 0.35 bar was set before each analysis, to remove any possible interfering compounds that could be present in the external airflow and to promote the cleaning of the sensitive materials of the sensors. After that, the gas static headspace of each vial (~25 mL) was collected by suction (vacuum pump) and delivered to the sensing unit, allowing the interaction, during 2.5 min, of the VOCs with the 9 MOS sensors, generating the respective resistive signal profiles, which were acquired each 4 s. Before pumping the sample’s gas headspace, each glass vial, previously inoculated with a single strain and a known number of CFUs, was placed inside the sampling unit during 13 min to allow reaching the desired temperature (~30 °C). The cleaning step was promoted during the sample heating time. Overall, two experimental designs were conducted. In the first, in order to evaluate the qualitative classification (i.e., discrimination) performance of the E-nose, glass vials (25 mL) with 7 mL of BHI agar medium, were inoculated with 100 µL of different diluted solutions obtained from an overnight culture of one of the four bacteria under study: dilutions of 10^−1^ to 10^−7^ for *E. faecalis* and dilutions of 10^−1^ to 10^−9^ for the other three bacteria. In the second kind of assay used for evaluating the quantitative performance of the E-nose to assess the number of CFUs of a single bacterium, sterile glass vials (25 mL) containing 7 mL of BHI agar medium were inoculated with 100 µL of different diluted solutions of an overnight grown bacteria culture with a known amount of CFUs. In this way, the vials were inoculated with dilutions of 10^−4^ to 10^−8^ for *E. faecalis* (from 1.2 to 1.2 × 10^6^ CFUs), and from 10^−5^ to 10^−9^ for the other bacteria (*E. coli*: 2.2 to 2.2 × 10^8^ CFUs; *P. aeruginosa*: 3.05 to 3.05 × 10^5^ CFUs and *S. aureus*: 1.2 to 1.2 × 10^6^ CFUs). After inoculation, the vials were closed with screw caps and incubated at 37 °C for 24 h, being only analyzed with the E-nose for those with visible bacterial growth.

#### 2.4.3. Data Acquisition, Feature Extraction and Signal Treatment

As previously described [32], the resistance signals (in ohms) generated due to the interaction between the VOCs emitted by each bacterium (*E. coli*, *P. aeruginosa*, *E. faecalis*, or *S. aureus*), grown in solid medium, and the nine MOS sensors, were acquired by a data logger (Agilent 34970A) and then recorded by the Agilent BenchLink Data Logger software. For each analysis and sensor, a total of 37–38 resistance values were recorded (signals acquired during 2.5 min each 4 s). In total, seven feature extraction methods [32,34] were applied to obtain a representative E-nose fingerprint for the volatile profile of the compounds emitted during the growth of each of the four bacteria under study: last response point (LP) acquired; integral of each E-nose signal response curve (INT); maximum resistance response (MAX); minimum resistance response (MIN); the sum of the response curve (SUM): the sum of all the resistance signals recorded during the analysis time-period; the average of the resistance signal curve (MEAN); and standard deviation (SD) of the response curve. In this context, for each independent sample a response database comprising 9 sensors × 7 feature extraction method, is generated, being the basis for the statistical analysis.

### 2.5. Statistical Analysis

Linear discriminant analysis (LDA) in combination with the simulated annealing (SA) algorithm was implemented as a supervised classification multivariate procedure to assess the E-nose predictive performance towards the discrimination of the four bacteria evaluated, based on the recorded resistance signals generated by the interaction between the sensors and the VOCs emitted by each bacterium, grown in solid medium. The classification potential was evaluated by determining the sensitivities (i.e., the correct classification percentages) and the specificities for the leave-one-out cross-validation (LOO-CV) variant. This CV variant was implemented since it is recommended for evaluating chemometric models in small sample sizes [35]. In total 40 independent samples (10 per type of bacterium) were used for training, and then for LOO-CV, being at each validation run, one sample removed for validation purposes and the other 39 to establish the model. Furthermore, for the training set, the classification was also checked graphically, by plotting the 2D graphs for the two most significant discriminant functions (DFs). Multiple linear regression (MLR) models, based on feature extraction parameters selected using the SA algorithm, were also developed. For this, the E-nose signals used were those generated when analyzing vials with solid medium, inoculated with pre-established known amounts (in CFU) of each of the four studied bacteria. The accuracy of the E-nose-MLRM was discussed based on the determination coefficients (*R*^2^) and the root-mean-square errors (RMSEs) for the training (10, 9, 6, and 7 samples for *E. faecalis*, *E. coli*, *S. aureus*, and *P. aeruginosa*, respectively) and LOO-CV procedures. The accuracy of the developed models was verified against the analytical conventional counting plate technique, being evaluated if the slope and intercept values of the regression line between the data for both approaches could be statistically assumed equal to those of a perfect line (i.e., 1 and 0, respectively), following the methodology previously described by the research team [29]. All statistical analyses were performed using the packages of the open-source statistical program R (RStudio 2021.09.0 Build 351) at a 5% significance level.

## 3. Results and Discussion

### 3.1. Evaluation of VOCs Emitted during the Bacterial Growth by HS-SPME-GC-MS

The VOCs emitted during the bacterium growth depend on the bacterial metabolism that is influenced by the culture media composition and nutrient sources. Thus, many bacterial VOCs have been identified and their amounts and profiles greatly depend on the culture medium, incubation time, and bacterial species and strains used, as contradictory detection/non-detection data is reported [5,6,9]. Background VOCs related to the growth media have also been reported [5].

Thus, as a first step, the VOCs emitted by the BHI un-inoculated medium were determined. Globally, 15 volatiles were identified (data not shown), and 10 of them were also emitted by at least one of the four bacteria evaluated, namely two alcohols (1-butanol and 1-nonanol), three pyrazines (2-ethyl-6-methyl-pyrazine, 3-ethyl-2,5-dimethylpyrazine, and trimethylpyrazine), three terpenes (camphene, D-limonene, and β-pinene), and two other compounds (2,4-thujadiene and indole). The volatiles were identified in both un-inoculated and inoculated (with a 10^−4^ dilution for *E. faecalis* and 10^−5^ dilution for the other bacterial strains) solid culture medium (i.e., BHI agar medium), after overnight incubation. Table 1 shows the amounts (in ng of each compound as IS equivalents) of the VOCs detected during the chromatographic analysis of the volatile head-space generated during the overnight growth of the two Gram-positive (*E. faecalis* and *S. aureus*) and the two Gram-negative (*E. coli* and *P. aeruginosa*) bacteria, after subtracting the respective amounts quantified in the head-space of the un-inoculated culture medium.

As can be seen from Table 1, VOCs belonging to 10 chemical classes were identified as the alcohols the most abundantly emitted by *E. coli*, *E. faecalis*, and *P. aeruginosa* during their growth in BHI solid medium, while esters were the predominant volatiles produced by *S. aureus* grown in the same culture medium. It should also be noticed that each bacterium had a specific profile of emitted VOCs, both in number of different volatiles identified as in the quantified amounts. In total, 17 VOCs were emitted during the growth of *E. faecalis*, followed by 14 VOCs for *E. coli*, 13 VOCs for *S. aureus* and only 10 VOCs for *P. aeruginosa*. As can also be inferred, some VOCs were specifically produced by only one of the four studied bacteria (*E. coli*: 1-nonanol, 2-tridecanone, 3-ethyl-2,5-dimethylpyrazine, and D-limonene; *P. aeruginosa*: (E)-1,4-undecadiene; *E. faecalis*: ethanol, 2-methylbutanoic acid, acetic acid, and methyl undecyl ether; *S. aureus*: 3-chloro-2-methyl-2-pentanol, α-phellandrene, trimethylpyrazine, and camphene), while others were common to two or more bacteria, although they were emitted, in general, in quite different amounts. These differences, in the emitted VOCs profiles and respective amounts generated by each bacterium growth, confirmed that VOCs can be used as bacterial identification biomarkers. However, it should be remarked that, for example, in this study, ethanol was only identified during the growth of *E. faecalis*, although according to the literature this alcohol is usually emitted by the four bacteria evaluated, being more abundant for *E. coli* and *S. aureus* compared to *E. faecalis* and *P. aeruginosa* [6]. Indole is usually associated in the literature with *E. coli* [4,5,6,7,8,9,10,11] but, in the present study, it was only identified for *P. aeruginosa* and *S. aureus*. On the other hand, (E)-1,4-undecadiene was only identified for *P. aeruginosa*, in-line with the literature data, according to which this alkene has been associated with *Pseudomonas* species, allowing its use as a biomarker for this species [36]. The observed differences may be tentatively attributed to the different bacterium strains, growth media and/or incubation conditions (time and temperature) used in the various studies as well as to the diverse chromatographic analysis conditions applied.

### 3.2. Bacterial Species Discrimination Using the E-Nose-MOS Lab-Made Device

The dataset comprising the resistance signals based feature extraction data generated by the E-nose sensors (9 sensors × 7 feature extraction variables, for each vial inoculated with different pre-established CFU of a single bacterium) was used to evaluate the potential application of the lab-made E-nose for discriminating the four bacteria under study, which were grown in solid agar medium (BHI agar).

**Figure 1:**
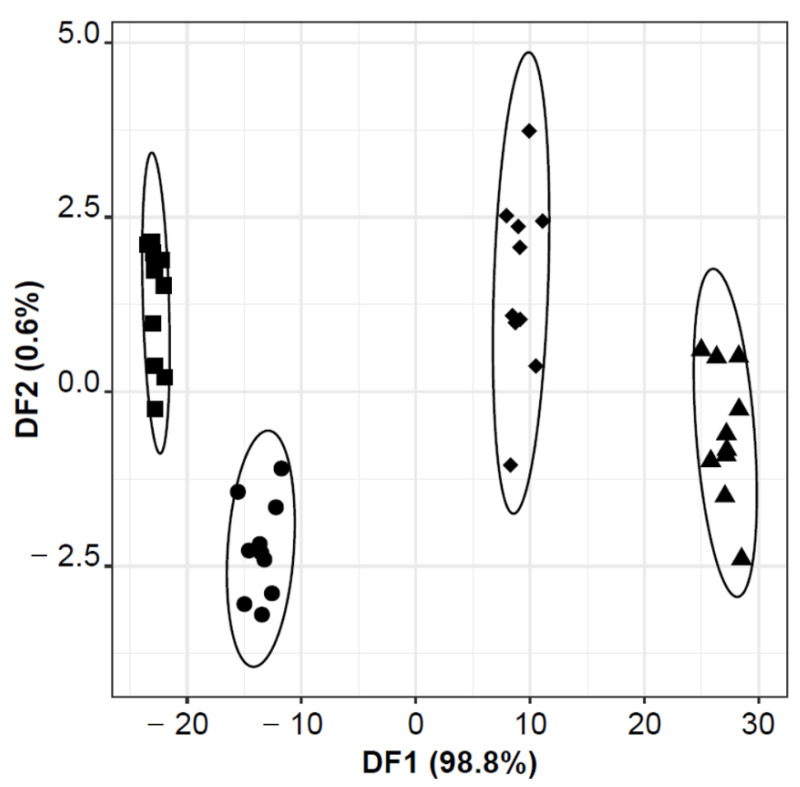
E-nose-MOS-LDA-SA model’s discrimination of the four studied Gram-positive and Gram-negative bacteria grown in BHI agar medium: (▲) *E. faecalis*; (♦) *S. aureus*; (■) *E. coli*; and, (●) *P. aeruginosa*. Classification model based on 25 feature extraction MOS signal parameters (S6_LP, S1_INT, S5_INT, S8_INT, S9_INT, S1_MAX, S2_MAX, S4_MAX, S6_MAX, S2_MIN, S4_MIN, S5_MIN, S6_MIN, S2_SUM, S3_SUM, S5_SUM, S6_SUM, S8_SUM, S9_SUM, S2_MEAN, S5_MEAN, S1_SD, S2_SD, S3_SD, and S6_SD).

Figure 1 shows that an E-nose-MOS-LDA-SA model could be established, based on 25 feature extraction parameters selected by the SA algorithm (S6_LP, S1_INT, S5_INT, S8_INT, S9_INT, S1_MAX, S2_MAX, S4_MAX, S6_MAX, S2_MIN, S4_MIN, S5_MIN, S6_MIN, S2_SUM, S3_SUM, S5_SUM, S6_SUM, S8_SUM, S9_SUM, S2_MEAN, S5_MEAN, S1_SD, S2_SD, S3_SD, and S6_SD), which first two DFs explained 99.4% of the data variability. The model enabled the full discrimination of the four bacteria (sensitivity and specificity of 100%, for the training/original grouped data). Moreover, it should be highlighted that, according to the 1st DF, it was possible to successfully discriminate the two Gram-positive bacteria (*E. faecalis* and *S. aureus*) from the two Gram-negative bacteria (*E. coli* and *P. aeruginosa*), located in the positive and negative regions, respectively. The satisfactory classification performance of each single bacterial species as well as between Gram-positive and Gram-negative bacteria, could be attributed to the observed differences, in the number and respective amount, of the VOCs emitted during the bacterial growth of each species (Table 1). As previously pointed out, the VOCs’ profiles established in this study by HS-SPME-GC-MS clearly pointed out the existence of volatiles emitted by only one of the four bacteria, which would generate different signal responses during the E-nose analysis, justifying the discrimination power of this MOS sensor device. It should be highlighted that, although not confirmed in the present study, other VOCs have been reported in the literature, as bacterial species’ biomarkers, namely isovaleric acid, or 2-methyl-butanal for *S. aureus*; 1-undecene, 2,4-dimethyl-1-heptane, 2-butanone, 4-methyl-quinazoline, hydrogen cyanide, or methyl thiocyanide for *P. aeruginosa*; and, methanol, pentanol, ethyl acetate, or indole for *E. coli* [6].

In which concerns the predictive performance, an overall correct classification of 90% was achieved for the LOO-CV procedure, with a global specificity of 91% (Table 2). As can be inferred, among the four bacteria, only *S. aureus* was not misclassified (sensitivity of 100%) but had the lowest specificity of (83%). On the contrary, one of the 10 assays with *E. coli* was misclassified as *S. aureus* (sensitivity and specificity of 90%); one *E. faecalis* was misclassified as *P. aeruginosa* (sensitivity of 90% and specificity of 100%); and finally, one sample of *P. aeruginosa* was incorrectly classified as *E. coli* and another as *S. aureus* (sensitivity of 80% and specificity of 89%).

The misclassifications observed, although in a low number (4 in 40 independent bacteria samples) can be attributed to the fact that some VOCs are emitted by different bacterial species, which mitigate their use as unique chemical fingerprints. Indeed, the VOCs profiles established in this study showed that several volatiles were emitted by two or more of the four bacteria under study, although in different amounts (e.g., 1-butanol, 1-pentanol, phenylethyl alcohol, phenol, isocetane, 1-undecene, isovaleric acid, undecane, methyl valerate, 2,5-dimethylpyrazine, 2-ethyl-6-methyl-pyrazine, β-pinene, 2,4-thujadiene, or indole). Additionally, a systematic review performed by Bos et al. [6] revealed that, for example, the four bacteria produce isopentanol, formaldehyde, methyl mercaptan, and trimethylamine, although not observed in the present study.

Nevertheless, it should be remarked that the discrimination rates (training and LOO-CV) are similar to the E-nose classification performances previously reported in the literature, in which sensitivities varied from 84 to 100%, allowing differentiating/discriminating several bacterial species, including *E. coli*, *E. faecalis*, *P. aeruginosa*, or *S. aureus*, among other pathogenic bacteria [1,2,15,16,17,18,19,20,21,22,23].

### 3.3. Quantification of Bacteria CFUs Using the E-Nose-MOS Lab-Made Device

As previously mentioned, few studies report the use of E-noses as semi-quantitative [23,26] or quantitative [27,28] tools to assess the levels of bacteria. In this sense, the present study also aimed to evaluate the quantitative performance of the lab-made E-nose-MOS for determining the number of each type of bacterium (i.e., CFUs) in the BHI agar medium. For that, MLRM were developed relating the decimal logarithmic of the CFUs (log_10_(CFU)) of each one of the four bacteria as a multiple linear function of the selected feature extracted parameters derived from the E-nose-MOS response towards the VOCs emitted by every single bacterium grown in solid medium. The predictive models’ performances were evaluated based on two goodness of fitting parameters (*R*^2^ and RMSE) for the LOO-CV variant. Table 3 shows the goodness of fitting data as well as the information regarding the concentration range studied and the number of parameters included in each developed MLRM, which were selected by the SA algorithm.

**Figure 2:**
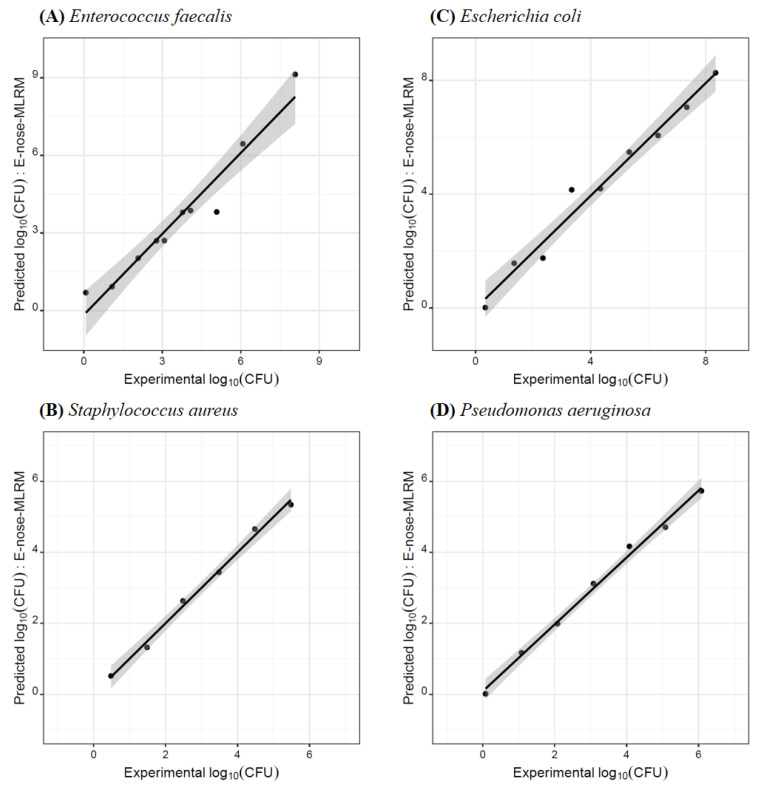
E-nose-MOS-MLR-SA model’s predictions (leave-one-out cross-validation) of the decimal logarithmic of the colony forming units (CFU), i.e., number of microorganisms, for the studied Gram-positive and Gram-negative bacteria grown in BHI agar medium. (**A**) *E. faecalis*: model based on 4 feature extraction MOS signal parameters (S9_INT; S9_MIN; S8_MEAN and S5_SD); (**B**) *S. aureus*: model based on 3 feature extraction MOS signal parameters (S5_INT, S4_SUM and S4_MEAN); (**C**) *E. coli*: model based on 3 feature extraction MOS signal parameters (S4_LP, S6_SUM and S9_MEAN); and (**D**) *P. aeruginosa*: model based on 3 feature extraction MOS signal parameters (S8_MAX; S9_MAX and S9_SD).

The satisfactory predictive (LOO-CV) values of *R*^2^ and RMSE (0.943 ≤ *R*^2^ ≤ 0.994 and 0.158 ≤ RMSE ≤ 0.602 log_10_(CFU)), which are confirmed by the visualization of Figure 2, support the use of the lab-made E-nose-MOS for quantifying the number of CFU of each one of the four bacteria studied. Indeed, based on the RMSE values, the number of CFUs initially inoculated in BHI agar medium, could be estimated after 24 h of incubation (at 37 °C) with an accuracy as low as 1 to 4 CFUs, in-line with the accuracies previously reported in the literature for quantitative assessment of bacteria in food samples [27,28].

The possibility of using the E-nose-MOS as an alternative routine tool for estimating the number of CFU grown in a solid medium was further checked following the methodology previously described by the research team [29], which is based on the XPT 90-210 French standard. For each bacterium, the decimal logarithmic of the CFU, predicted by the E-nose-MOS-MLRM were plotted versus the decimal logarithmic of the experimental number of CFU, determined by counting plate technique, and the respective parameters (slope and intercept values) of the single regression trend line were calculated. The regression analysis allowed us to verify if the slope and intercept values were statistically equal to one and zero, respectively, which would correspond to a perfect linear fit. Table 4 shows the parameters of the single linear regressions (*R*^2^, slope and intercept values and the respective 95% confidence intervals, CI) for the LOO-CV procedure. The results clearly demonstrate that, at 5% significance level, the slope and intercept values were statistically equal to the expected theoretic values (i.e., the slope CI included the value 1; and, the intercept CI contained the value zero). Thus, it was confirmed that the lab-made E-nose-MOS device combined with MLRMs could be implemented as a fast, green, and non-invasive tool to estimate the number of CFUs of each of the four bacteria studied inoculated separately in BHI agar medium, based on the sensors’ response towards the VOCs emitted by each individual strain during a 24 h growing period in a solid synthetic medium.

## 4. Conclusions

The study carried out highlighted the feasibility of applying a self-built electronic nose prototype, comprising metal oxide semiconductor sensors, as a qualitative-quantitative tool for the identification and monitoring of four different bacteria. The designed device allowed the discrimination of the odors emitted by Gram-negative (*E. coli* and *P. aeruginosa*) and Gram-positive (*E. faecalis* and *S. aureus*) bacteria, grown overnight in a solid culture medium. However, it should be highlighted that the satisfactory predictive classification performance (sensitivities ranging from 80 to 100% and specificities varying between 83 to 100%) was achieved for the leave-one-out cross-validation variant, which could be slightly overoptimistic, being required an external validation in the future. Moreover, the olfactory-sensor device allowed assessing the number of colony-forming units, (i.e., the number of microorganisms) of each of the four bacteria studied, grown separately in BHI solid culture medium, with the respective root mean square errors as low as 1 to 4 colonies. Once again, this accuracy was obtained for the leave-one-out cross-validation variant and thus, the satisfactory quantitative results should be considered with caution. Nevertheless, the performed study can be seen as a proof of concept that the electronic nose is a fast, non-invasive and green alternative/complementary strategy to the conventional analytical approaches. Finally, the promising and reliable classification and quantitative performances achieved with the electronic nose strengthen the hypothesis of applying the proposed sensing-chemometric approach to the bacterial growth analysis in solid food samples, although the influence of environmental conditions and the impact arising from the complexity and diversity of real samples matrices must be considered.

## Figures and Tables

**Table 1:** Identified volatile compounds and respective average amounts (ng of compound as internal standard equivalents), for the *E. coli*, *P. aeruginosa*, *E. faecalis*, or *S. aureus*, grown overnight in BHI solid medium after incubation with a 10^−4^ dilution for *E. faecalis* and 10^−5^ dilution for the other bacterial strains.

Volatile Compounds	Gram-Negative Bacteria		Gram-Positive Bacteria	
	*E. coli*	*P. aeruginosa*	*E. faecalis*	*S. aureus*
Alcohols				
1-Butanol	33.82	nd	51.68	nd
1-Nonanol	5.42	nd	nd	nd
1-Pentanol	53.96	95.53	869.56	51.09
3-Chloro-2-methyl-2-pentanol	nd	nd	nd	11.11
Ethanol	nd	nd	695.93	nd
Phenylethyl alcohol	15.46	nd	23.30	nd
Aldehydes				
Phenol	6.19	nd	6.87	nd
Alkanes				
Isocetane	5.11	5.21	5.55	5.41
Alkenes				
(E)-1,4-Undecadiene	nd	70.09	nd	nd
1-Undecene	nd	32.00	23.71	43.71
Carboxylic acids				
2-Methylbutanoic acid	nd	nd	13.63	nd
Acetic acid	nd	nd	29.40	nd
Isovaleric acid	nd	43.40	37.17	nd
Undecane	9.89	11.45	16.63	7.22
Esters				
Methyl valerate	nd	70.17	nd	114.72
Ketones				
2-Tridecanone	7.92	nd	nd	nd
Phellandrenes				
α-Phellandrene	nd	nd	nd	6.81
Pyrazines				
2,5-Dimethylpyrazine	38.62	46.01	48.88	46.87
2-Ethyl-6-methyl-pyrazine	0.52	nd	0.79	nd
3-Ethyl-2,5-dimethylpyrazine	1.11	nd	nd	nd
Trimethylpyrazine	nd	nd	nd	0.59
Terpenes				
Camphene	nd	nd	nd	1.50
D-Limonene	0.74	nd	nd	nd
β-Pinene	2.56	4.78	4.71	3.08
Others				
2,4-Thujadiene	0.52	nd	1.47	0.59
E-7-Dodecen-1-ol acetate	nd	nd	11.67	nd
Indole	nd	2.97	nd	32.75
Methyl undecyl ether	nd	nd	19.82	nd

nd: not detected.

**Table 2:** Confusion matrix for the internal validation classification (LOO-CV) performance of the E-nose-MOS-LDA-SA model concerning the discrimination of the four studied bacteria (*E. faecalis*, *S. aureus*, *E. coli*; and *P. aeruginosa*) grown in BHI agar medium.

Actual Bacterium		Predicted Bacterium				Total	Sensitivity
		Gram-Negative		Gram-Positive			
		*E. coli*	*P. aeruginosa*	*E. faecalis*	*S. aureus*		
Gram-negative	*E. coli*	9	0	0	1	10	90%
	*P. aeruginosa*	1	8	0	1	10	80%
Gram-positive	*E. faecalis*	0	1	9	0	10	90%
	*S. aureus*	0	0	0	10	10	100%
Total		10	9	9	12	40	90%
Specificity		90%	89%	100%	83%	91%	---

**Table 3:** Predictive performance (LOO-CV) of the MLRM developed based on selected feature extraction parameters derived from the interaction of the E-nose-MOS sensors with the VOCs emitted by each one of the four bacteria studied: *E. coli*, *P. aeruginosa*, *E. faecalis*, and *S. aureus* (number of each bacterium, i.e., contents in log_10_(CFU)).

Microorganism		Concentration Range (log_10_(CFU)) ^a^	Selected Extracted Feature Parameters ^b^	Goodness of Fitting Parameters ^c^	
				*R* ^2^	RMSE (log_10_(CFU))
Gram-negative	*E. coli*	[0.342, 8.342]	S4_LP; S6_SUM; S9_MEAN	0.978	0.436
	*P. aeruginosa*	[0.079, 6.079]	S8_MAX; S9_MAX; S9_SD	0.995	0.174
Gram-positive	*E. faecalis*	[0.079, 8.079]	S9_INT; S9_MIN; S8_MEAN; S5_SD	0.943	0.602
	*S. aureus*	[0.484, 5.484]	S5_INT; S4_SUM; S4_MEAN	0.994	0.158

^a^ Experimental concentration range in log_10_(CFU): 9 independent levels for *E. coli*; 7 independent levels for *P. aeruginosa*; 10 independent levels for *E. faecalis*; and 6 independent levels for *S. aureus*. ^b^ Feature extracted parameters from the E-nose-MOS response, selected by the SA algorithm and included in each MLRM. ^c^ *R*^2^: determination coefficient; RMSE: root mean square error.

**Table 4:** Parameters of the single linear regressions established between decimal logarithmic of the CFU predicted (LOO-CV) by the E-nose-MOS-MLM and the decimal logarithmic of the CFU experimentally determined by the conventional plate counting technique: coefficient of determination (*R*^2^); slopes, intercept values and respective confidence intervals (CI) at 95%.

Microorganism		*R* ^2^	Slope	Slope CI	Intercept (log_10_(CFU))	Intercept CI (log_10_(CFU))
Gram-negative	*E. coli*	0.978	0.990	[0.857, 1.124]	−0.016	[−0.690, 0.658]
	*P. aeruginosa*	0.995	0.943	[0.863, 1.022]	0.082	[−0.211, 0.375]
Gram-positive	*E. faecalis*	0.943	1.044	[0.834, 1.254]	0.167	[−1.060, 0.726]
	*S. aureus*	0.994	0.998	[0.893, 1.103]	0.004	[−0.358, 0.366]

## Data Availability

The data presented in this study are available on request from the corresponding author.
